# β‐Sitosterol—Dietary sources and role in cancer and diabetes management

**DOI:** 10.1002/fsn3.4380

**Published:** 2024-09-11

**Authors:** Karthikeyan Adhimoolam, Anjana Sureshbabu, Elena Smirnova, Pandiyan Muthuramalingam, Cat Tuong Do Thi, Kalaiselvi Senthil, Taesun Min

**Affiliations:** ^1^ Subtropical Horticulture Research Institute Jeju National University Jeju South Korea; ^2^ Department of Animal Biotechnology, Bio‐Resources Computing Research Center, Sustainable Agriculture Research Institute (SARI) Jeju National University Jeju South Korea; ^3^ Division of Horticultural Science, College of Agriculture and Life Sciences Gyeongsang National University Jinju Republic of Korea; ^4^ Department of Biochemistry, Biotechnology and Bioinformatics Avinashilingam Institute for Home Science and Higher Education for Women Coimbatore India

**Keywords:** food crops, human nutrition, plant sterols, β‐sitosterol

## Abstract

β‐Sitosterol is a major bioactive constituent and the most abundant phytosterol in nuts, seeds, and vegetable oils. It is structurally similar to cholesterol, except for the addition of the ethyl group. The primary benefit of β‐sitosterol is that it lowers the body's absorption of low‐density lipoprotein, or “bad” cholesterol. Research efforts to date and information from the available literature have demonstrated that β‐sitosterol has many pharmacological benefits to improve human health; it effectively prevents heart diseases, cancer, and diabetes. To date, many investigations on β‐sitosterol have been conducted in in vitro and in vivo studies. There are considerable research gaps because there are almost no clinical studies to examine the safety and effectiveness of β‐sitosterol for various human diseases. This review aims to discuss the dietary sources and variations of β‐sitosterol in food crops and how it can successfully prevent cancer and diabetes, including the mechanism underlying these benefits. In addition, we also discuss the research gaps and provide our perspective on future research to propose β‐sitosterol as a nutraceutical candidate to prevent human diseases.

## Graphical abstract

This review aims to discuss the dietary sources and variations of β‐sitosterol in food crops and how it can successfully prevent cancer and diabetes, including the mechanism underlying these benefits. We also discuss the research gaps and provide our perspective on future research to propose β‐sitosterol as a nutraceutical candidate to prevent human diseases.

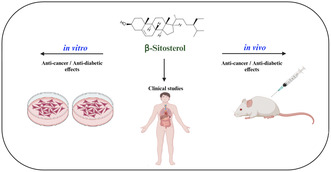

## 1 INTRODUCTION

Phytosterols, also referred to as plant sterols or stanol esters, are biologically active compounds found in plant cell membranes with a chemical structure resembling the cholesterol obtained from mammalian cells. There are no double bonds present in the sterol ring of plant stanols, in contrast to plant sterols, which are unsaturated sterol compounds possessing a double bond at the C‐5 position (Law, 2000). Phytosterols can exist in their free or bound form in combination with fatty acids or carbohydrates as esterified phytosterols or glycosides (Ogbe et al., 2015). Humans cannot synthesize phytosterols; therefore, they can be taken in recommended amounts with the diet and transferred to human blood vessels and tissues. Therefore, phytosterols are important micronutrients in human diets. Functional foods that have been enriched with them or nutraceuticals may be taken for phytosterol supplementation. To date, more than 250 phytosterols have been identified in the free and esterified form (Cohn et al., 2010; Moreau et al., 2002). Among them, the most prevalent phytosterol is β‐sitosterol, and it abundantly exists in several lipid‐rich plant‐based foods (i.e., nuts, seeds, and vegetable oils) (Chanioti et al., 2021; Gupta, 2020). In the view of plants, β‐sitosterol is regarded as an important chemical substance because it supports the survival of plants. Although its role in the chloroplast and cytoplasm is still unclear, numerous studies have demonstrated that β‐sitosterol stabilizes the cell membrane. Taking β‐sitosterol‐enriched foods or supplements is a good dietary treatment for lowering cholesterol levels (Bin Sayeed & Ameen, 2015). β‐Sitosterol has a long history of use in pharmaceutical products and is widely regarded as a safe and viable nutritional supplement with no harmful side effects. β‐Sitosterol has various biological actions, including the prevention of cardiovascular diseases (Hu, 2003; Retelny et al., 2008), having anticancer (Bao et al., 2022; Choi et al., 2003; Vundru et al., 2013), anti‐inflammatory (Loizou et al., 2010; Nirmal et al., 2012), antioxidant (Gupta et al., 2011), antidiabetic (Babu & Jayaraman, 2020) properties, as well as acting as precursors of vitamin D and hormones (Nguyen et al., 2022).

Globally, cancer and diabetes are very common diseases that have a major influence on health, and their incidence continues to increase yearly. Epidemiological data indicate that people with diabetes have a high chance of being affected by cancers. Despite so many clinical studies, the available preclinical studies, such as in vitro, in vivo, and epidemiological studies, detailed how effectively β‐sitosterol contributes to the deterrence and treatment of cancer (Ditty & Ezhilarasan, 2021; Shin et al., 2018) and diabetes (Babu et al., 2020; Ponnulakshmi et al., 2019). Like other natural compounds, β‐sitosterol has systemic biological activity and multitarget effectiveness. The protective effects against cancer and diabetes were mainly activated by apoptosis and cell cycle arrest, regulating oxidative stress, modulating immunity and inflammation, promoting metabolic reprogramming, and combating drug resistance. Taking into account the above‐mentioned facts, this review provides the details of the dietary sources and variations of β‐sitosterol, including how it can successfully combat cancer and diabetes. Additionally, we debate the research gaps and our viewpoint on future research to recommend β‐sitosterol as a potential nutraceutical candidate for disease management.

## 2 A BRIEF ACCOUNT OF β‐SITOSTEROL'S CHEMICAL STRUCTURE AND SYNTHESIS

Similar to cholesterol, phytosterols have a tetracyclic core structure, although there are differences in the type of substitution at C24 and whether a double bond is present at C22.

**FIGURE 1:**
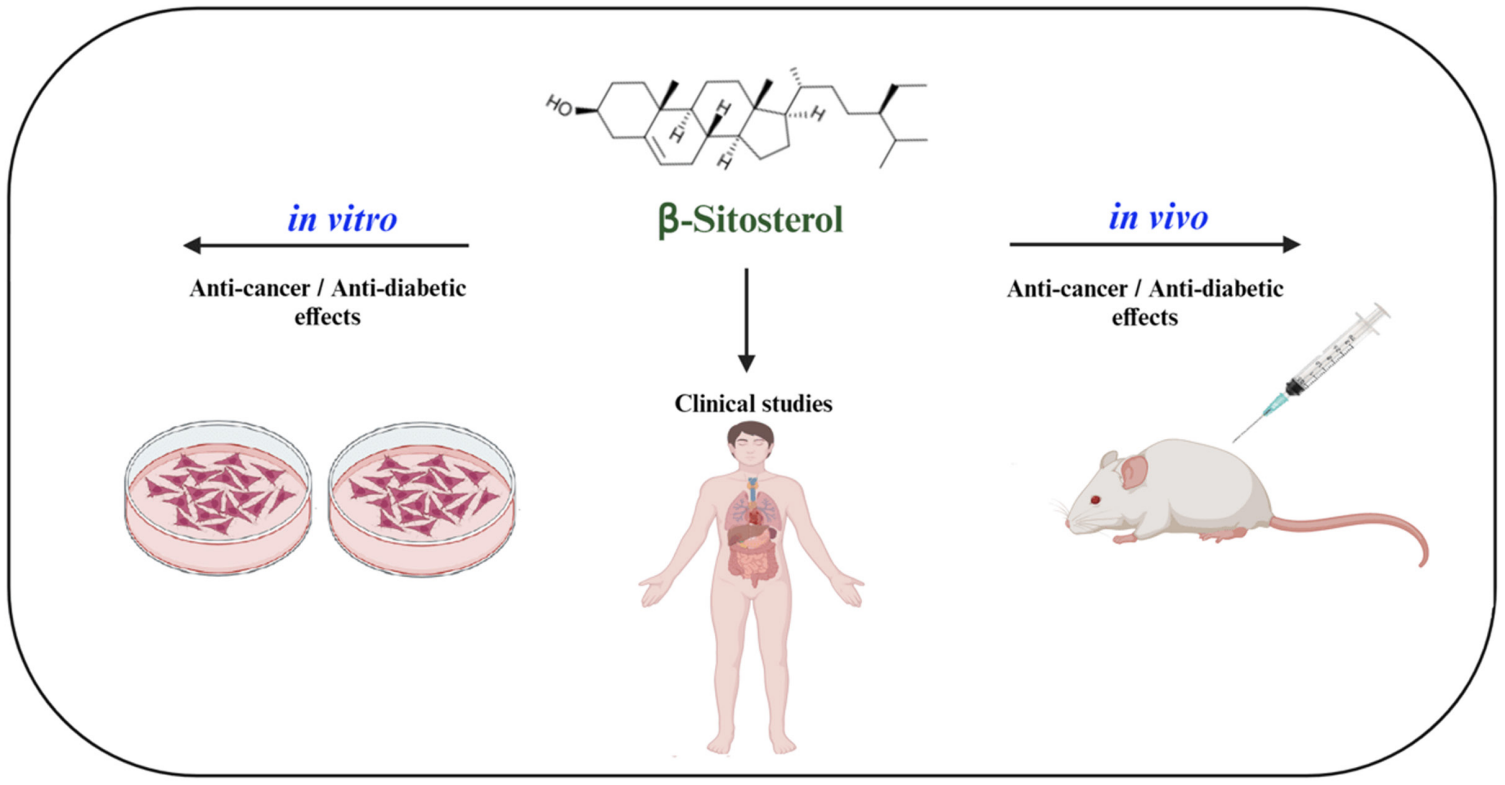
The chemical structure and benefits of β‐sitosterol.

**FIGURE 2:**
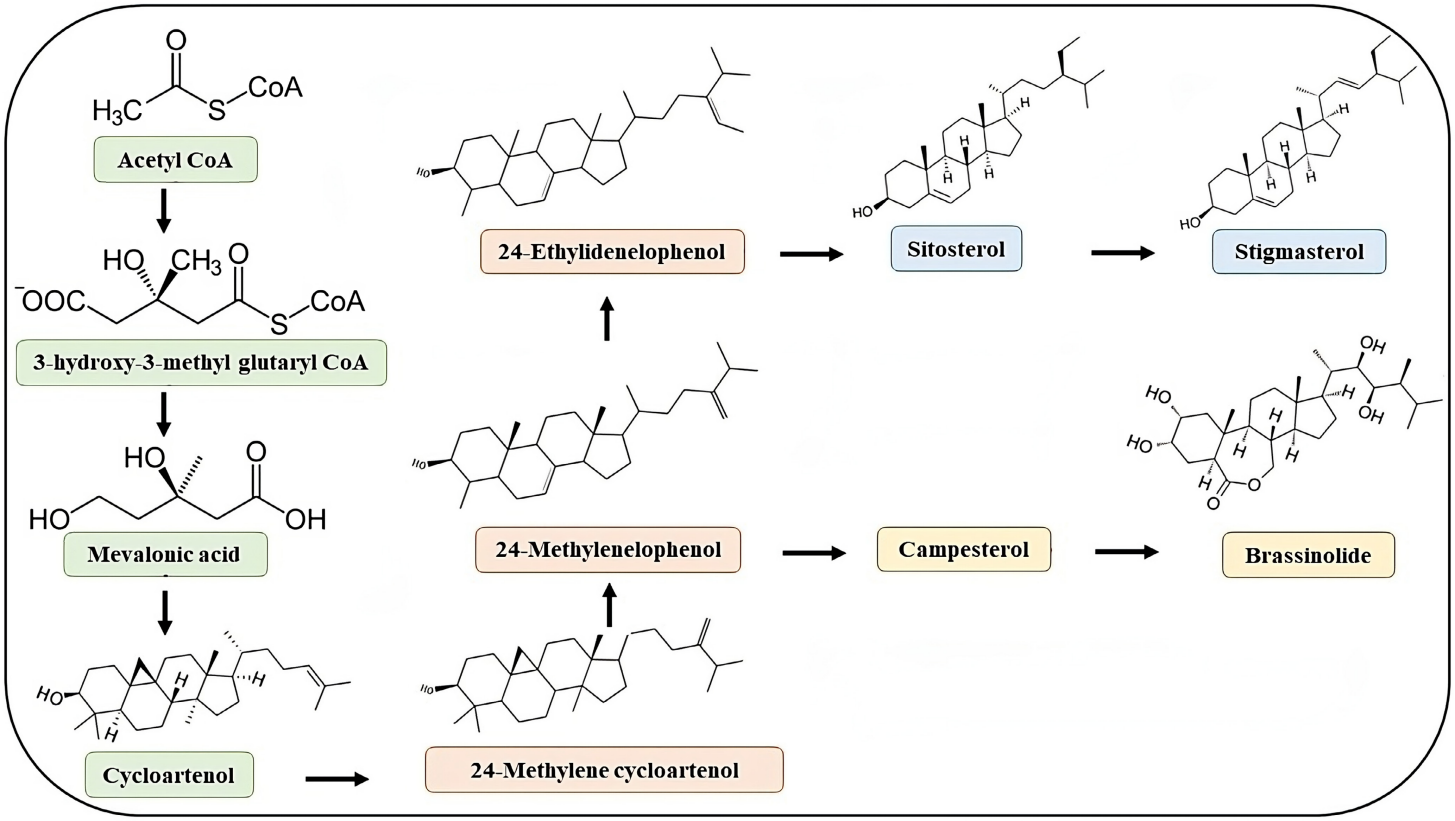
Schematic illustration for the biosynthesis of β‐sitosterol.

β‐Sitosterol is the primary phytosterol present in the majority of plant diets. β‐Sitosterol (C29H50O), molecular weight 414.71 g/mol, is an unsaturated sterol with one double bond in a sterol ring structure (Chanioti et al., 2021; Pegal, 1997; Ryan et al., 2006). A steroidal molecule with optical activity, β‐sitosterol's chemical name [(3S,8S,9S,10R,13R,14S,17R)‐17‐[(2R,5R)‐5‐ethyl‐6‐methylheptan‐2‐yl]‐10,13‐dimethyl 2,3,4,7,8,9,11,12,14,15,16,17‐dodecahydro‐1H‐cyclopenta [a] phenanthren‐3‐ol] reflects its optical activity (Gupta, 2020). Table 1 shows the β‐sitosterol's chemical composition. There are some sterols that are similar to the β‐sitosterol structure. Campesterol, a sterol that structurally resembles β‐sitosterol but lacks an ethyl group at position C24 (replaced by methyl group), and stigmasterol (It is an unsaturated sterol with two double bonds: one in the sterol ring and one in the side chain) (Klingberg et al., 2008; Ryan et al., 2006). Stigmastanol is a saturated sterol with a sterol ring structure and side chain.β‐Sitosterol is not completely synthesized in two different routes; it is produced from pure stigmasterol. In the first route from the hydrogenation of side chain Δ22–23 alkene, β‐Sitosterol was produced along with completely saturated stigmastanol and various amounts of stigmasterol. In another route, discerning hydrogenation happens, followed by Δ5–6 alkene protection to generate the cyclopropylcarbinyl ether. This route synthesizes highly pure β‐sitosterol. To produce 5–6 alkene and C3 alcohol, this method should proceed by hydrogenating the Δ22–23 double bond and solvolysis of the cyclopropane (Hang & Dussault, 2010; Khripach et al., 2005). With 13C‐labeling patterns, lipids and sterols are biosynthesized in membrane biogenesis. Several researchers have demonstrated that deoxyxylulose and mevalonate pathways are involved in the β‐sitosterol synthesis (De‐Eknamkul & Potduang, 2003; Neelakandan et al., 2010; Ohyama et al., 2009). The precise mechanism associated with the production of β‐sitosterol synthesis involves cycloartenol. However, it varies depending on the organism. Farnesyl diphosphate (FPP) is produced from cycloartenol biosynthesis via joining one isopentenyl diphosphate and two dimethylallyl diphosphate molecules. Triterpene (squalene) is formed when two FPP molecules are attached tail to tail. Squalene reacts with an intermediate 2,3‐oxidosqualene to form cycloartenol, which is then produced through cyclization. A key precursor for the biosynthesis of sterols is cycloartenol, which is generated from squalene by the enzyme cycloartenol synthase. Following that, cycloartenol is changed into 24‐methylene cycloartanol by methylation. After that, it goes through several enzymatic reactions to produce 24‐methylenelophenol. The transformation of 24‐methylenelophenol into 24‐ethylenelophenol produces fucosterol and then β‐sitosterol, which is the precursor to avenasterol. The detailed chemical structure and synthesis of β‐sitosterol are given in Figures 1 and 2.

**TABLE 1:** Summary of physicochemical properties of β–sitosterol.

S. No.	Characteristics	
1	Formula	C_29_H_50_O
2	Num. heavy atoms	30
3	Molecular weight	414.7 g/mol
4	Fraction Csp3	0.93
5	Num. aromatic heavy atoms	0
6	Num. rotatable bonds	6
7	H‐bond acceptors' count	1
8	Num. H‐bond count	1
9	Molar refractivity	133.23
10	Topological polar surface area	20.2 Å^2^
11	Number of violations (nVio)	1
12	Melting temperature	140°C
13	Bioavailability score	0.55
14	Gastrointestinal absorption	Low
15	Blood–brain barrier (BBB) permeant	No
16	Nuclear receptor ligand	0.73
17	Enzyme inhibitor	0.51

## 3 DIETARY SOURCES AND VARIATION OF β‐SITOSTEROL

In this section, we discuss the available literature about the variation of β‐sitosterol content in well‐known sources, such as vegetables and fruits. β‐Sitosterol can be found in plant tissues, such as seeds, leaves, and rhizomes. It has undergone various chromatographic techniques for isolation and purification. The β‐sitosterol content in major vegetables and fruits ranges from 44% to 86% and 72% to 92%, respectively (Piironen et al., 2003). In vegetable oils, the proportion of β‐sitosterol in total sterols ranges from 38% to 61% (Lima & Block, 2019). In vegetables, it was found that β‐sitosterol accounts for 40.8 mg/100 g in cauliflower, 34.5 mg/100 g in broccoli, 14.0 mg/100 g in carrot, 10.4 mg/100 g in cabbage, 9.9 mg/100 g in ginger, 8.7 mg/100 g in garlic, 10.4 mg/100 g in lettuce, 5.4 mg/100 g in spinach, 3.8 mg/100 g in cucumber, 6.6 mg/100 g in sweet potato, 6.2 mg/100 g in onion, 2.9 mg/100 g in tomato, 3.8 mg/100 g in radish, 1.8 mg/100 g in potato, and 2 mg/100 g in eggplant (Han et al., 2008). In grain legumes, it was found that β‐sitosterol accounts for 41.4 mg/100 g in peas, 19.4 mg/100 g in cowpea, and 7.5 mg/100 g in soybean sprouts (Han et al., 2008). It was found that β‐sitosterol accounts for 12.5 mg/100 g in apple, 19.6 mg/100 g in orange, 9.3 mg/100 g in banana, 10.9 mg/100 g in strawberry, 12.0 mg/100 g in pineapple, 21.1 mg/100 g in tangerine, 19.4 mg/100 g in mango, 13.4 mg/100 g in kiwifruit, 8.6 mg/100 g in papaya, 12.0 mg/100 g in pineapple, 11.6 mg/100 g in peach, 11.0 mg/100 g in cherry, and 11.6 mg/100 g in apricot (Han et al., 2008). In vegetable oil, it was found that β‐sitosterol accounts for 189.12 ± 42.40 mg/100 g in peanut oil, 166.03 ± 43.62 mg/100 g in soybean oil, 394.11 ± 146.74 mg/100 g in rapeseed oil, 322.73 ± 85.81 mg/100 g in sesame oil, 152.05 ± 58.58 mg/100 g in olive oil, 50.09 ± 13.71 mg/100 g in camellia oil, 539.93 ± 160.08 mg/100 g in corn oil, 170.91 ± 26.18 mg/100 g in sunflower oil, 157.79 ± 24.37 mg/100 g in flaxseed oil, 735.17 ± 185.99 mg/100 g in rice bran oil, 165.23 ± 69.07 mg/100 g in walnut oil, 258.71 ± 18.45 mg/100 g in peony oil, and 146.63 ± 14.67 mg/100 g in grapeseed oil (Yang et al., 2019). The composition of total phytosterols and β‐sitosterol in different vegetables, fruits, and vegetable oils is summarized in Table 2.

**TABLE 2:** Composition of total phytosterols and β‐Sitosterol in vegetables, grain legumes, fruits, and vegetable oils (mg/100 g).

S. No.	Category	Items	Total phytosterols	β‐Sitosterol
1	Vegetables	Broccoli	40.9	34.5
2		Cauliflower	42.8	40.8
3		Carrot	19.4	14.0
4		Cabbage	13.6	10.4
5		Cucumber	7.3	3.8
6		Coriander leaf	18.7	9.3
7		Eggplant	2.9	2
8		Garlic	11.2	8.7
9		Ginger	15.0	9.9
10		Onion	7.4	6.2
11		Romaine lettuce	30.9	16.7
12		Potato	3.6	1.8
13		White radish	5.1	3.8
14		Spinach	10.6	5.4
15		Sweet potato	9.9	6.6
16		Tomato	6.1	2.9
17	Grain legumes	Cowpea	29.8	19.4
18		Pea	53.7	41.4
19		Soybean	14.2	7.5
20	Fruits	Apricot	13.4	11.6
21		Banana	12.6	9.3
22		Cherry	11.3	11
23		Kiwi fruit	17.5	13.4
24		Mango	24.4	19.4
25		Orange	24.2	19.6
26		Papaya	16.9	8.6
27		Peach	13.7	11.6
28		Pineapple	16.6	12.0
29		Strawberry	11.8	10.9
30		Tangerine	25.5	21.1
31	Vegetable oils	Camellia oil	142.64 ± 50.86	50.09 ± 13.71
32		Corn oil	990.94 ± 240.76	539.93 ± 160.08
33		Flaxseed oil	466.73 ± 60.65	157.79 ± 24.37
34		Grapeseed oil	273.80 ± 38.85	146.63 ± 14.67
35		Olive oil	288.02 ± 92.60	152.05 ± 58.58
36		Peanut oil	319.75 ± 76.15	189.12 ± 42.40
37		Peony oil	367.19 ± 42.13	258.71 ± 18.45
38		Rapeseed oil	893.84 ± 237.77	394.11 ± 146.74
39		Rice bran oil	1891.82 ± 500.76	735.17 ± 185.99
40		Sesame oil	637.60 ± 180.59	322.73 ± 85.81
41		Soybean oil	355.67 ± 91.85	166.03 ± 43.62
42		Sunflower oil	253.25 ± 46.60	170.91 ± 26.18
43		Walnut oil	272.04 ± 107.41	165.23 ± 69.07

*Note*: Data related to total phytosterols and β‐sitosterol in vegetables, grain legumes, fruits (Han et al., 2008), and vegetable oils (Yang et al., 2019) summarized from previous studies.

## 4 THE STORY OF VARIOUS β‐SITOSTEROL FORMULATIONS

Limited water solubility, absorption, bioavailability, crystallization at room temperature, body temperature, and high amount of daily doses (up to 3 g/day) are the major issues interrelated to the oral supplement of β‐sitosterol. These issues mainly limited the β‐sitosterol production and the progress of clinical trials. The efficient incorporation of β‐sitosterol in drug preparation is a difficult task. Therefore, many researchers have attempted to improve β‐sitosterol absorption using different administration routes and synthesizing novel formulations or combinations with other drugs. It is known that the therapeutic effectiveness of different natural compounds, including β‐sitosterol, is increased by the use of nanoparticle drug delivery systems (Bilia et al., 2017; Evtyugin et al., 2023). Researchers have investigated the benefits of combination with cyclodextrin, liposomes, electrospun nanofibers, solid lipid nanoparticles, polymers, self‐emulsion drug delivery system, and nanostructured lipid carriers on therapeutic effectiveness by increasing oral absorption of β‐sitosterol (Abdou et al., 2019; Afzal et al., 2022; Andima et al., 2018; Awad et al., 2000; Imanaka et al., 2008; Prabahar et al., 2022; Soleimanian et al., 2020). Due to the dose‐dependent increase in bioavailability that only occurred in the colon, β‐sitosterol‐β‐D‐glucoside nanoparticles (β‐sit‐β‐D‐gluco‐NPs) improved the colon‐specific absorption of fluorescein isothiocyanate (FITC)–dextran 4400 (FD‐4). It suggests that β‐sit‐β‐D‐gluco‐NP could be associated with endocytosis through glucose residue of β‐sit‐β‐D‐gluco (Nakamura et al., 2003). Imanaka et al. demonstrated that the β‐sitosterol liposomes increased the activity of natural killer (NK) cells and reduced the colonies of B16BL6 melanoma cells in the lungs of experimental mice (Imanaka et al., 2008). Amina et al. (2017) successfully encapsulated β‐sitosterol in polyurethane by sol–gel electrospinning method and examined the therapeutic benefits in the embryonic mouse NIH 3T3 fibroblast cell line model. By using the grinding method, β‐sitosterol‐2‐hydroxypropyl‐β‐cyclodextrin (Sit‐HP‐β‐CD) was synthesized with a 1:2 molar ratio of Sit to HP‐β‐CD. The results of the in vitro study showed that Sit‐HP‐β‐CD was more effective than β‐sitosterol in lowering intracellular lipid accumulation and the expression levels of fatty acid synthase (FAS) and proliferator‐activated receptor‐gamma (PPARγ) in 3T3‐L1 cells, indicating that the complex formed with Sit and HP‐β‐CD improved the inhibitory effect on adipogenesis (Yu et al., 2018). Andima and coworkers prepared β‐sitosterol‐loaded polyglycolic acid (PLGA) and polyethylene glycol–polylactic acid (PEG–PLA) nanoparticles (β‐Sit‐PLGA [215.0 ± 29.7 nm] and β‐Sit‐PEG–PLA [240.6 ± 23.3 nm]) by the simple emulsion–solvent evaporation method. Results showed that PLGA was a better polymer for encapsulating β‐sitosterol than PEG–PLA, with better cellular uptake. Also, β‐sitosterol's antiproliferative effectiveness against Michigan Cancer Foundation 7 (MCF‐7) and MDA‐MB‐231 (M.D. Anderson and metastasis breast cancer) cells was increased according to the dose by being encapsulated in PLGA nanoparticles (Andima et al., 2018). Abdou and coworkers synthesized the lipid–polymer hybrid nanoparticles (LPHNPs) encapsulated with β‐sitosterol and showed the hepatoprotective effect on carbon tetrachloride (CCl4)‐induced hepatotoxicity in rats (Abdou et al., 2019). β‐Sitosterol solid lipid nanoparticles were prepared by double emulsion–solvent displacement approach, and they showed good loading capacity, entrapment efficiency, and particle size distribution and improved in vitro release characteristics. This β‐sitosterol nanoformulation ameliorates complete adjuvant‐induced arthritis in rats through nuclear factor‐kappa B (NF‐кB) and heme oxygenase 1/nuclear factor erythroid 2‐related factor 2 (HO‐1/Nrf‐2) pathway (Zhang et al., 2020). Raj (2020) successfully used β‐sitosterol as a reducing agent in silver nanoparticle (AgNP) synthesis. The outcome of the study revealed that β‐sitosterol‐assisted silver nanoparticles (BSS‐SNPs) assist as a prospective candidate drug for human hepatocellular cancer cells; these nanoparticles activated Nrf‐2 and caused mitochondrial death via oxidative stress. Recently, β‐sitosterol alginate/chitosan nanoparticles (β‐sito‐Alg/Ch/NPs) showed higher cytotoxicity to breast cancer cells because of better bioavailability and antioxidant capacity than the normal β‐sito‐suspension (Afzal et al., 2022). Researchers made biodegradable polymeric microneedles using chitosan as the long‐acting supplement of β‐sitosterol loaded with a nanostructured lipid carrier, and they also showed that it is useful to treat androgenic alopecia and other androgen‐linked diseases (Prabahar et al., 2022). The complex interplay among the epidermal growth factor receptor (EGFR) and hepatocyte growth factor receptor (HGFR/MET) resulted in dysregulation and was associated with cancer pathogenesis and therapeutic resistance. β‐Sitosterol conjugation with biodegradable polymers [β‐sitosterol (BS) with superparamagnetic iron oxide nanoparticles (SPIONs) (BS‐conjugated SPIONs (BS–S)), poly(N‐isopropylacrylamide) (PNIPAM) (BS‐conjugated SPIONs–PNIPAM (BS–SP)), PEG, and PNIPAM (BS‐conjugated SPIONs–PEG–PNIPAM (BS‐SPP)) polymers] increased the drug encapsulation efficiency. It may be used as the likely treatment for deterring EGFR and MET receptor‐expressed cancer cells (MCF‐7 human breast cancer), HepG2 (liver cancer), and NCIH 460 (non‐small cell lung cancer) (Ilangovan et al., 2023). On the other hand, the supplement of β‐sit‐β‐D‐gluco via nasal route enhanced the bioavailability by up to 90.7%. Verapamil was more readily absorbed via the nasal mucosa when given concurrently with β‐sitosterol or β‐sit‐β‐D‐gluco. It is hypothesized that β‐sit‐β‐D‐gluco promotes drug absorption by increasing the fluidity of the mucosal membrane and facilitating Ca++ inflow from extracellular sources (Maitani et al., 2000). Studies were conducted on suspensions that had various amounts of β‐sitosterol, oil, and water. Three different types of β‐sitosterol have been shown to exist: crystals that are anhydrous, hemihydrated, and monohydrated crystals. In place of platy‐like anhydrous crystals, monohydrated needle‐shaped crystals were made when water was added. Particles with a needle shape created structured suspensions that thinned under shear. High supersaturation and subsequent small crystal formation were caused by a high sterol content (Christiansen et al., 2002).

## 5 PREVENTIVE EFFECTS OF β‐SITOSTEROL ON DIFFERENT TYPES OF CANCERS

**FIGURE 3:**
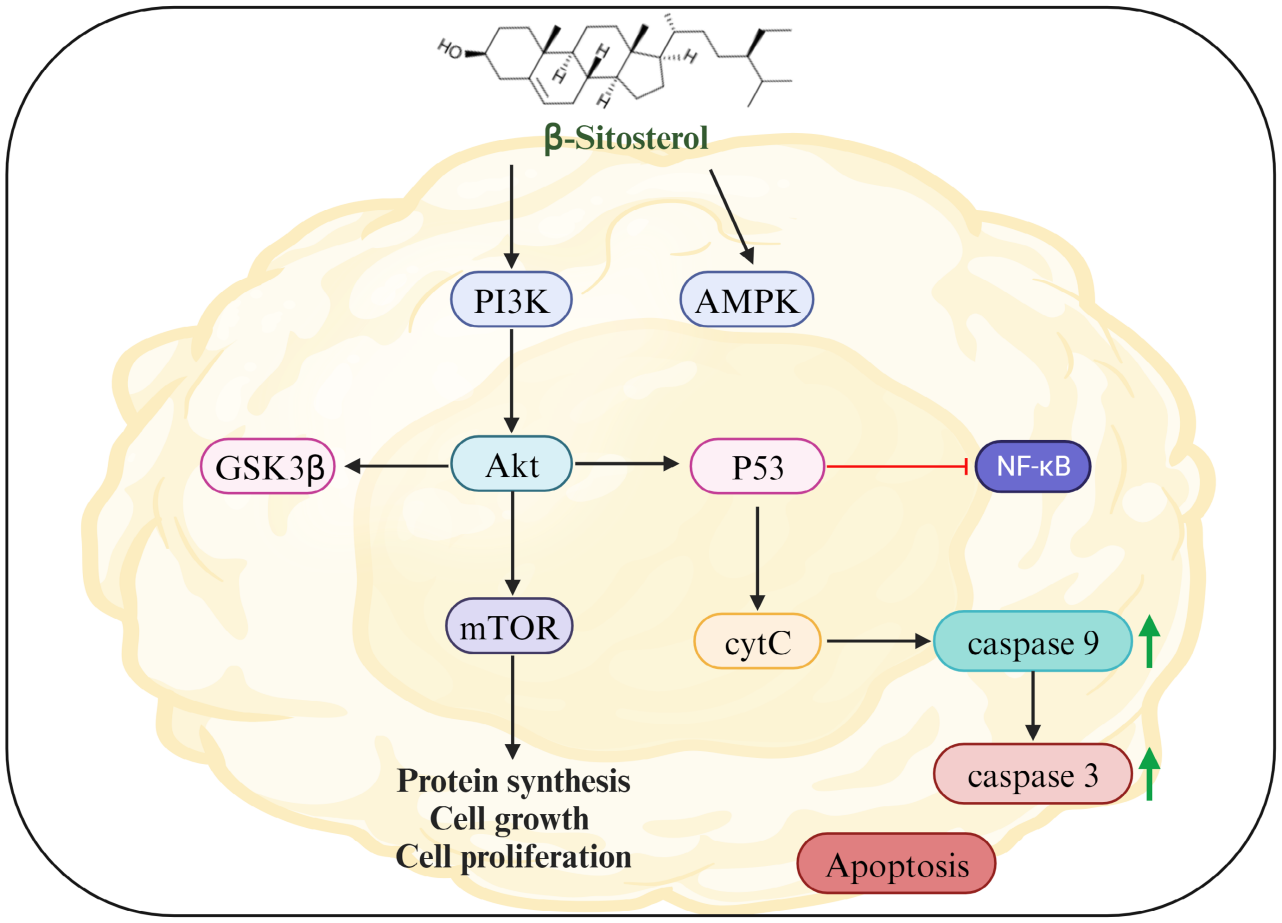
β‐Sitosterol mechanism of action and intervention targets in cancer.

Many researchers have detailed that β‐sitosterol effectively obstructs cancer cell proliferation, and this activity is linked to the cell cycle arrest, promotion of apoptosis cell death, and sphingomyelin cycle (Wang et al., 2023). The existing evidence showed a remarkable ability of β‐sitosterol and its derivatives to induce apoptosis in different cell lines by promoting the apoptosis via modulating the FAS levels and Caspase‐8 activity, phosphorylation of extracellular signal‐regulated kinase (ERK), and p38 mitogen‐activated protein kinase (p38 MAPK), preventing the cancer cell growth at low doses while having no cytotoxic effects on normal cells (Awad et al., 2003, 2005, 2007; Choi et al., 2003; Moon et al., 2007, 2008; Von Holtz et al., 1998). Moreover, it exerts the G2/M phase cell cycle arrest, reduces free radicals’ production, and regulates the antioxidant enzyme activity during pathogenesis (Jayaprakash et al., 2007; Moon et al., 2008). The underlying mechanism of β‐sitosterol against cancer is presented in Figure 3. The anticancer effects of β‐sitosterol have been extensively studied in different cancers, such as prostate, pancreatic, breast, gastric, colon, liver, and renal cancers. β‐Sitosterol anticancer effects against various cancers are summarized in Table 3 and discussed in detail below.

**TABLE 3:** Summary of research on the effects of β‐sitosterol on cancer treatment, both in vitro and in vivo.

S. No.	Type of cancer	Cell line/animal model	Dose, duration, and route of administration	Main findings	References
1	Prostate cancer	LNCaP cells	16 μM	Cancer cell death, sphingomyelin cycle activation induces cancer cell apoptosis and elevates the protein phosphatase 2A's activity	von Holtz et al. (1998), Awad et al. (2000)
		MIA‐PaCa‐2 and BXPC‐3 cells	250/L	Suppressed cell growth and triggered programmed cell death, enhanced cell cycle arrest at G0/G1 phase, inhibited NF‐kB activity and AKT/GSK‐3β pathway, elevated Bax expression (Pro‐apoptotic protein), and reduced Bcl‐2 expression (Anti‐apoptotic protein), decreasing the expression of epithelial–mesenchymal transition markers	Cao et al. (2020)
		PC‐3, DU‐145	50 and 80 μM; 40 and 70 μM	Inhibition of cellular migration, decrease in cellular viability, and induction of apoptosis	Pradhan et al. (2019)
		PC‐3	25, 50 and 100 μM	Cell growth was suppressed, and apoptosis was induced, decreasing the rate of prostate cells through EMT signaling molecules	Reddy et al. (2022)
2	Pancreatic cancer	MIA‐PaCa‐2 and BXPC‐3	250 μM/L	Induced apoptosis decreases migration and invasion, downregulating EMT markers’ expression and AKT/GSK‐3β signaling pathway	Cao et al. (2020)
		BxPC‐3	0, 10, 50, and 100 μg/mL	Suppresses pancreatic cancer cell growth, inhibits migration of pancreatic cancer cells, and inhibits mTOR activation through IGFBP3–PI3K pathway	Jang et al. (2023)
3	Breast cancer	MDA‐MB‐231 cells	16 μM	Suppressed the cancer cell growth, triggered a cytotoxic effect, leading to the induction of apoptosis, and the activity of tyrosine kinase was reduced	Awad et al. (2000)
		MCF‐7 cells	10 μM	Suppressed the cancer cell growth, an elevated Caspase‐8 activity was observed	Chai et al. (2008)
		MDAMB‐231 and MCF‐7 cells	16 μM	Stimulation of serine palmitoyltransferase activity resulted in de novo synthesis of ceramide	Awad et al. (2008)
		MCF‐7 cells	10, 20, 30, 40, and 50 μM	Increased drug release pattern, improved bioavailability, and prevented the generation of radicals	Afzal et al. (2022)
		MDA‐MB‐231 cells	OA 25 + β 4 μM	Triggers apoptosis that inhibits the Notch receptors in breast cancer	Shukla et al. (2023)
4	Colon cancer	HT116 cells	7.5 and 20 mM	Cancer cell inhibition is based on the dose‐dependent manner, and the treatment activated Caspases‐3 and ‐9, which led to the cleavage of poly (ADP‐ribose) polymerase through proteolysis. Additionally, the expression of Bcl‐2 and Bax proteins was decreased and increased	Choi et al. (2003)
		COLO 320 DM cells/Wistar rats	10–20 mg/kg; 16 weeks; pellet supplement	The growth of cells was suppressed, and apoptosis was induced, reactive oxygen species were eliminated, levels of proliferating cell nuclear antigens and β‐catenin were decreased	Baskar et al. (2010)
		HT‐29 cells	8 and 16 μM	The growth of cells was inhibited, and the levels of membrane, sphingomyelin, and cholesterol were reduced by 50% and 26%, respectively, modulating the signaling pathway linked to membrane phospholipids	Awad et al. (1996)
		HT‐115 cells	0.1, 0.2 mg/dL	Inhibition of colon cancer cell proliferation and activation of Bax, Caspase‐3, and Caspase‐9	Salamatullah et al. (2021)
		HT‐116 cells	7.5 and 20 μM	Colon cancer cell inhibition based on the dose‐dependent proteolytic cleavage, induction of Bax, and activation of Caspase‐3 and Caspase‐9	Choi et al., 2003
		HT‐115 cells	0.1 and 0.2 mg/dL	Induced cellular and nuclear damage, suppression of oxidative stress, inhibited colon cancer cell proliferation	Salamatullah et al. (2021)
		HCT116 cells	75 and 125 μg/mL	Apoptosis induction, suppression of invasion, and anoikis induction through the EGFR/Akt pathway in HCT116 colorectal cancer cells	Kawk et al. (2021)
5	Gastric cancer	SGC‐7901 cells	20 μmol/L	The proliferation was inhibited, apoptosis was increased, and a reduction in expression of pAKT, mTOR, and p‐PI3K	Sun et al. (2019)
		Adenocarcinoma AGS cells/ AGS xenograft mouse models (BALB/c)	0, 10, 25,50, and 100 μg/mL	Cancer cell growth was arrested, and the expressions of PTEN and Hsp90 proteins were elevated and reduced, respectively	Shin et al. (2018)
6	Renal cancer	Male albino rats	20 mg/kg, oral administration	Stimulation of programmed cell death and suppression of cell growth. The expressions of cyclin‐D1, proliferating cell nuclear antigen (PCNA), vascular endothelial growth factor (VEGF), and Bcl‐2 (anti‐apoptotic protein) were reduced, while expressions of caspases and pro‐apoptotic Bax protein were upregulated	Sharmila and Sindhu (2017)
7	Liver cancer	Huh7 and HepG2 cells	1.25, 2.5,5, 10, 20, and 40 μg/mL	Initiation of cytotoxic activity and Caspase‐3 and caspase‐9 were activated	Vo et al. (2020)
		HepG2 cells	0.6, 1.2 mM/mL	Elevated cytosolic expression of cytochrome c and activation of Caspase‐3	Ditty and Ezhilarasan (2021)
		HepG2 cells	20–60 μg/mL	Accelerates the regulation of Bax, p^53^, Caspase‐3, and Caspase‐9	Pal et al. (2022)

### 5.1 Prostate cancer

Lymph node carcinoma of the prostate (LNCaP) cells were treated with β‐sitosterol delivered by a cyclodextrin vehicle. The findings showed that β‐sitosterol suppressed 24% of cell growth, while the 4‐fold apoptosis process was increased, which resulted in cell rounding, and ceramide production was elevated to 50%. Moreover, the administration of β‐sitosterol caused cell death by promoting the sphingomyelin cycle. However, the treatment did not impact the prostatic acid phosphatase (PAP) or prostate‐specific antigen (PSA), while the total acid phosphatase content was greater than before up to 7 days posttreatment (Von Holtz et al., 1998). Additionally, β‐sitosterol exhibited a lower level of inhibition on the growth of human prostate cancer cell lines (Jourdain et al., 2006). Treatment with β‐sitosterol significantly increases E‐cadherin (CDH1) expression levels in PC‐3 and DU‐145 human prostate cancer cell lines. Decreased CDH1 expression is associated with cancer initiation and progression, and impairment of this gene augments the invasive and metastatic abilities, consequently hastening the progression of the tumor (Pradhan et al., 2019). Downplay of E‐cadherin (CDH1) and epithelial–mesenchymal transition (EMT) play critical roles in facilitating tumor invasion and metastasis (Zhao et al., 2021). β‐Sitosterol exhibits anticancer properties in prostate cells, reducing cell proliferation by modulating EMT signaling molecules (Reddy et al., 2022). EMT is linked with tumor development, invasiveness, metastasis, and resistance to treatments. This process endows cells with migratory and invasive traits, which cancer cells can choose during metastasis (Huang et al., 2022). Protein phosphatase 2A (PP2A) activation is considered to play a role in the sphingomyelin cycle activation by β‐sitosterol therapy. Nevertheless, the minimum but significant increase in the activity of phospholipase D, along with supplementation of β‐sitosterol, suggests that other mechanisms may regulate this pathway. These mechanisms may include the β‐sitosterol integration into cell membranes, altering fluidity and consequently inducing the membrane‐bound enzymes, namely phospholipase D activation (Mitra et al., 2023).

### 5.2 Pancreatic cancer

β‐Sitosterol exhibited significant suppression of pancreatic cancer cell line development through diverse cellular mechanisms, such as cell division (G0/G1 phase) arrest, decreased proliferation, and apoptosis. It also reduced the activity of NF‐κB while simultaneously increasing and decreasing the expressions of Bcl‐2‐associated X protein (Bax) and B‐cell lymphoma‐2 (Bcl‐2) protein, respectively (Khan et al., 2022). Additionally, the research revealed that β‐sitosterol had a preventive effect on invasion and migration while inhibiting the protein kinase B/glycogen synthase kinase 3 (AKT/GSK‐3) signaling pathways and EMT markers. The synergistic action of β‐sitosterol and gemcitabine on the growth rate of BXPC‐3 and MIAPaCa‐2 human pancreatic cancer cells revealed a substantial effect (Cao et al., 2020). Notably, in a controlled environment, the mixture of β‐sitosterol and gemcitabine considerably decreased the growth of pancreatic cancer in the xenograft mice model. Therefore, synergism of β‐sitosterol with gemcitabine may be an effective strategy for pancreatic cancer therapy. There is a significant upregulation of insulin‐like growth factor (IGF) and IGF receptors on the surface of pancreatic cancer cells, facilitating cancer progression (Deng et al., 2022). The interaction between IGF and its receptor triggers the activation of phosphatidylinositol‐3‐kinase (PI3K)/Akt and ERK signaling pathways, ultimately leading to cancer cell survival, proliferation, and migration (Thomas & Radhakrishnan, 2020). In contrast, insulin‐like growth factor‐binding protein (IGFBP) inhibits IGF‐associated signaling pathways by disrupting the IGF‐to‐IGF receptor interaction (Jin et al., 2020). Jang et al. (2023) reported that treatment with a methanolic extract of *Mychonastes* sp. promoted upregulation of insulin‐like growth factor‐binding protein 3 (IGFBP3) expression, the most prevalent IGFBP, while concurrently suppressing the activation of the PI3K–mTOR (mammalian target of rapamycin) signaling cascade. It implies that β‐sitosterol in the methanolic extract of *Mychonastes* sp. may significantly impact the regulation of the IGFBP–PI3K pathway in pancreatic cancer cells (Jang et al., 2023).

### 5.3 Breast cancer

Researchers have studied the impact of β‐sitosterol on cells associated with breast cancer. In one study involving the MDA‐MB‐231 cell line, supplementing 16 μM of β‐sitosterol for three consecutive days accelerated apoptosis by up to sixfold (Awad et al., 2000). The MCF‐7 cell proliferation was observed to be dose‐dependently inhibited by *Cyrtandra cupulata* derived β‐sitosterol, possibly through caspase‐induced apoptosis (Chai et al., 2008). Moreover, β‐sitosterol induced G2/M phase arrest of the cell cycle in MDA‐MD‐231 cells and exhibited a 70% growth retardation effect. Furthermore, β‐sitosterol increases Caspase‐8 activity and the amount of Fascia, reducing tumor cell growth. Also, β‐sitosterol elevates the Bax protein‐to‐Bcl‐2 protein ratio, which may induce apoptosis (Awad et al., 2007; Rauf et al., 2021). A derivative of β‐sitosterol, named β‐sitosterol‐d‐glucoside, has also been found to activate proteases and induce apoptosis in MDA‐MB‐231 and MCF‐7 cells. Oral therapy with β‐sitosterol‐d‐glucoside resulted in decreased tumor growth by hampering the PI3K–Akt pathway, reducing tumor‐associated antigens CEA, CA125, and CA153 levels, and elevating microRNA‐10a (miR‐10a) expression (Xu et al., 2018). In addition, β‐sitosterol was found to decrease the levels of cyclin D1 and cyclin‐dependent kinase 4 (CDK4) while increasing the expressions of tumor suppressors (cyclin‐dependent kinase (CDK) interacting protein (Cip)/kinase inhibitor protein (Kip) family of proteins), ultimately inducing apoptosis (Vundru et al., 2013). The results of these studies imply that β‐sitosterol could be potentially employed to prevent and treat breast cancer. Afzal et al. (2022) comprehensively delineated nanoantioxidant therapy based on β‐sitosterol‐derived nanoparticles (sito‐Alg/Ch/NPs), using alginate/chitosan as the nanoparticle matrix. The outcomes of in vitro and in vivo investigations substantiated that these nanoparticles displayed higher cytotoxicity due to increased drug release profiles, improved bioavailability, and enhanced antioxidant activity in breast cancer (Afzal et al., 2022). Studies indicate that tumor cells’ progression, survival, and development involve various developmental pathways, including Wnt, Hedgehog, and Notch. Among these signaling pathways, the Notch signaling pathway is highly conserved in many species, including humans and sea urchins. During development and morphogenesis, it is essential for cell differentiation, cell survival, cell proliferation, stem cell renewal, and determination of cell fate (Edwards & Brennan, 2021). Shukla et al. (2023) studied the synergistic effect of oleanolic acid and β‐sitosterol on the Notch pathway. This study revealed that the combination of oleanolic acid and β‐sitosterol is a promising therapeutic for the Notch pathway. Additionally, this combination treatment can target other aberrantly activated pathways, including Akt, which plays a crucial role in breast carcinoma (Shukla et al., 2023). Hence, the synergistic effects of oleanolic acid and β‐sitosterol may potentially serve as inhibitors of the Notch and Akt pathways, presenting a potential therapeutic option for breast cancer patients.

### 5.4 Gastric cancer

According to research, β‐sitosterol caused apoptosis and reduced cell viability in AGS human gastric adenocarcinoma cells. Apoptotic cells were spotted using Caspase‐3/7 activity, Annexin V, and the MitoPotential test (Shin et al., 2018). Additionally, phosphorylated adenosine monophosphate (AMP)‐activated protein kinase (p‐AMPK) and phosphatase and tensin homolog (PTEN) expression were increased due to the presence of β‐sitosterol in the liver cells, and AMPK was identified as a possible PTEN upstream regulator. A 2D (two‐dimensional) gel electrophoresis was subjected to protein identification associated with β‐sitosterol therapy, and heat shock protein 90 (Hsp90) protein levels were reduced. β‐Sitosterol reduced tumor development in AGS xenograft mice models without inducing damage by altering the PTEN, AMPK, and Hsp90 proteins. β‐Sitosterol and its anticancer mechanism in gastric cancer cells may be connected to the PI3K/AKT/mTOR pathway‐induced autophagy (Shin et al., 2018; Sun et al., 2019). Almost all types of tumors exhibit abnormal cell cycle activity, leading to tumor cell proliferation (Suski et al., 2021). Targeting specific cell cycle components is a potential strategy for combating cancer. In vitro studies have shown that β‐sitosterol inhibits the growth of AGS cells, a type of gastric cancer cell, by inducing apoptosis and arresting the cell cycle in the S phase, which may be attributed to the regulation of the p53 pathway (Zhong et al., 2022).

### 5.5 Colon cancer

Several studies have investigated the effects of β‐sitosterol on colon cancer cells. In one study, β‐sitosterol efficiently obstructed the HT‐29 cell growth than the lipids or control treatments. The β‐sitosterol supplementation to the diet can reduce the membrane cholesterol levels and decrease membrane sphingomyelin levels by half (Awad et al., 1996; Khan et al., 2022). Treatment with β‐sitosterol also resulted in changes to the fatty acid content of certain phospholipids but did not affect sphingosine production or the activity of phospholipase C (Awad et al., 1998). β‐Sitosterol's antiproliferative machinery was further delineated in another investigation. It found that it inhibited cell proliferation based on the concentration‐dependent manner in HCT116 human colorectal carcinoma cells and induced apoptotic cell death. β‐Sitosterol treatment also increased tumor suppressor p53 accumulation and cyclin‐dependent kinase (CDK) inhibitor p21 levels (Choi et al., 2004). Other studies found that the addition of β‐sitosterol concentration‐dependently lowered the crypt multiplicity in rats and the number of aberrant crypts treated with 1,2‐dimethylhydrazine (DMH) and suppressed oxidative stress genes in colon cancer cells as well as pro‐tumorigenic inflammation (Baskar et al., 2010). The β‐sitosterol‐mediated silver nanoparticles (AgNPs) were also found to cause cytotoxicity in HT‐29 cells in a concentration‐dependent manner and increased p53 expression (Shathviha et al., 2021). The bioactive substances in *Cassia alata* (L.) extract, notably cyclotrisiloxane and β‐sitosterol, may obstruct the proliferation of colon cancer cells (HT‐115 cells). This inhibition is achieved by suppressing the tumor growth‐promoting immune axis and activating the mitochondria‐dependent apoptotic pathway (Salamatullah et al., 2021). The epidermal growth factor receptor (EGFR) plays a main function in the induction of anoikis (Song et al., 2021). Caveolin‐1 is activated in response to EGFR activation, which suppresses anoikis and encourages tumor metastasis. Additionally, the activation of proto‐oncogene tyrosine‐protein kinase (Src), a signaling transducer, consecutively activates Akt, thus facilitating the proliferation of cancer cells and tumors (Yang et al., 2018). Since the stimulation of this pathway upsurges anoikis resistance, suppressing both EGFR and Akt is essential to induce anoikis. Kawk et al. (2021) examined that *Scaphium affine* extract, which comprises physiological compounds such as β‐sitosterol, exhibits potential anticancer properties in colorectal (colon) cancer by obstructing the signaling pathways EGFR and AKT.

### 5.6 Renal cancer

In a study published in Cancer Prevention Research and Treatment, the presence of apoptotic markers, cyclin D1, VEGF, and proliferating cell nuclear antigen (PCNA), was assessed by performing the regression of renal carcinogenesis. The alterations in the tumor cells were measured through various immunological techniques, including blotting, quantitative polymerase chain reaction (qPCR), and immunohistochemistry (Sharmila & Sindhu, 2017). The investigation discovered that administering oral β‐sitosterol prior to exposure to a kidney carcinogen considerably enhanced the expression of all markers and improved the histological characteristics in patients with kidney cancer (*p* < .05) (Sharmila & Sindhu, 2017).

### 5.7 Liver cancer

β‐Sitosterol exhibited anticancer properties, including liver cancer. In a study by Vo et al. (2020), β‐sitosterol and β‐sitosterol‐glucoside from *Indigofera zollingeriana* Miq were investigated for their anti‐hepatocellular‐cancer activity. The study found that both compounds had significant cytotoxic effects on Huh7 and HepG2 cells, inducing cell cycle arrest and apoptosis. In addition, the compounds could inhibit the HepG2 cell migration and invasion. The study suggests that β‐sitosterol and β‐sitosterol‐glucoside are therapeutic molecules to combat liver cancer (Vo et al., 2020). Ditty and Ezhilarasan (2021) demonstrated that β‐sitosterol administration triggers apoptosis in hepato‐and prostate‐carcinoma cells by stimulating reactive oxygen species (ROS) generation. Recent advances in oncological research indicate that intracellular ROS production caused by oxidative stress is a common mechanistic pathway for inducing both cytotoxicity and apoptosis in cancer cells (Rohit Singh & Ezhilarasan, 2020; Vairavel et al., 2020). Several research investigations have shown that silver nanoparticles (AgNPs), regardless of their mode of synthesis, can generate ROS within cells and consequently induce cytotoxic effects in various cancer cell lines, including those of the breast, lung, colon, cervix, prostate, pancreas, and liver (Khader et al., 2020; Zou et al., 2020). Furthermore, Raj (2020) reported that the β‐sitosterol‐assisted silver nanoparticles (BSS‐SNPs) facilitated intracellular ROS accumulation in HepG2 cells. BSS‐SNPs exhibited promising cytotoxic potential and triggered apoptosis through the intrinsic mitochondrial pathway (Raj, 2020). It proposes that BSS‐SNPs may be a plausible drug candidate for treating hepatocellular carcinoma.

## 6 PLAUSIBLE ANTIOXIDANT β‐SITOSTEROL; HOW IT MANAGES DIABETES BY REGULATING OXIDATIVE STRESS

Oxidative stress and diabetes (Type 1 and Type 2)‐associated conditions have been widely studied. Mounting results from in vitro, in vivo, and clinical studies describe a direct connection among hyperglycemia, oxidative stress, and diabetic complications (Asmat et al., 2016; Maritim et al., 2003; Piconi et al., 2003). In response to raised blood glucose levels, the mitochondrial electron transport chain overproduces reactive oxygen species (ROS). Increased ROS activity is observed in cellular components, altering DNA and protein and lipid peroxidation (Giacco & Brownlee, 2010). Free radical production in diabetes is greatly influenced by glucose oxidation, non‐enzymatic protein glycation, and the ensuing oxidative breakdown of glycated proteins. Remarkably, the increase in free radicals and a following deterioration in antioxidant defense mechanisms result in free radical damage to cellular organelles and enzymes, an upsurge in lipid peroxidation, and the development of insulin resistance (Rochette et al., 2014; Stadler, 2013). These negative effects of oxidative stress could encourage the growth of issues related to diabetes. Several studies showed that hyperglycemia targets and disturbs several biochemical pathways linked to ROS production, including activation of polyol pathways, glucose oxidation, and the advanced glycation end‐products (AGEs) (Giacco & Brownlee, 2010; Maritim et al., 2003).

**FIGURE 4:**
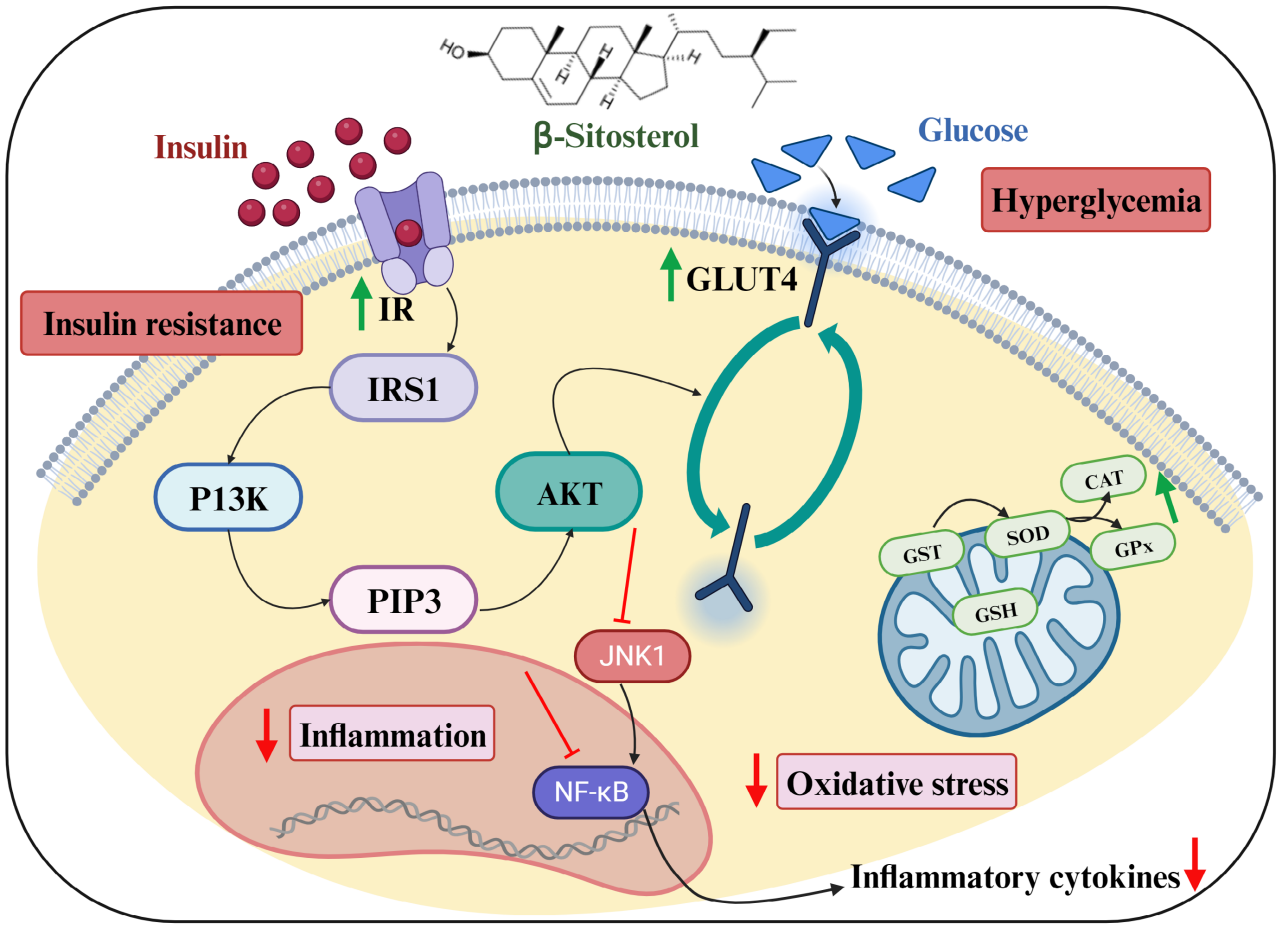
The underlying mechanism of β‐sitosterol in diabetes management.

Diabetes progresses more rapidly when oxidative stress is present; β‐sitosterol has received attention for its antioxidant properties, which function both physically and chemically as membrane stabilizers and moderate radical scavengers. The underlying mechanism of β‐sitosterol against diabetes is presented in Figure 4. Several studies have confirmed that β‐sitosterol is useful in treating diabetes; they are outlined and discussed here. In normal and streptozotocin (STZ)‐induced diabetic rats, Gupta et al. (2011) investigated the effects of a 21‐day treatment with β‐sitosterol (10, 15, and 20 mg kg, p.o.) on blood, serum, and tissue biochemical characters. Glycated hemoglobin (HbA1c), blood glucose, and nitric oxide (NO) all decreased at all three doses in a concentration‐reliant manner, whereas serum insulin levels simultaneously increased. Additionally, pancreatic antioxidant levels were elevated under treatment with β‐sitosterol dosages, while thiobarbituric acid‐reactive compounds were concurrently decreased. Ponnulakshmi et al., 2019 detailed the β‐sitosterol antidiabetic potential by in silico and in vivo analyses using the adipose tissue of a high‐fat diet (HFD) and sucrose‐induced type‐2 diabetic experimental rats. The major findings from their study were that oral administration of β‐sitosterol at a dose of 20 mg/kg body weight per day during 30‐day trials resulted in anti‐hyperlipidemic and anti‐hyperglycemic activities. In high‐fat diet‐induced type‐2 diabetic rats, these activities are regulated by insulin signaling molecules, such as insulin receptor (I.R.) and glucose transporter isoform 4 (GLUT4), which also normalize the metabolic profile, oxidative stress indicators, and antioxidant enzyme levels. In another study from Akilarooran et al., 2019, in comparison to the control group, type‐2 diabetic mice group livers had considerably increased amounts of lipid peroxidation (LPO), hydroxyl radical (OH*), and hydrogen peroxide (H_2_O_2_). β‐Sitosterol supplement effectively decreased the H_2_O_2_, OH*, and LPO. Chandran et al. (2019) detailed that in type‐2 diabetic rats given HFD and fructose induction, β‐sitosterol dominates the modulation of adiponectin and leptin circulation levels. Gumede et al., 2020 confirmed that an oral supplement of β‐sitosterol (20 mg/kg) protected the metabolic dysfunction caused by the supplement of a high‐fructose diet in female rats. In high‐fat diet‐ and streptozotocin‐induced diabetic rats, supplementation with β‐sitosterol (15 mg/kg body wt/day for 30 days) significantly reduced plasma glucose. Further evaluation of insulin resistance and glycosylated hemoglobin levels using the homeostatic model of insulin showed an upsurge in insulin and hemoglobin levels and peroxisome proliferator‐activated receptor c (PPARc) and GLUT4 expression at protein levels in insulin‐dependent tissues.

Additionally, β‐sitosterol prevents the body from losing weight and ingesting excessive amounts of food and liquids (Ramalingam et al., 2020). β‐Sitosterol (20 mg/kg b.wt) was supplied to type‐2 diabetic rats for 30 days; β‐sitosterol supplement elevates the lipolysis, decreases the proinflammatory adipokines (leptin and resistin) secretion, and shows an upsurge in the anti‐inflammatory adipokines’ (adiponectin) secretion. As a result, the inhibitor of nuclear factor‐κB (IκB) kinase (IKK)/NF‐κB and Jun N‐terminal kinase (JNK) signaling pathways in the adipose tissues are downregulated, and the production of pro‐inflammatory cytokines (TNF‐α and IL‐6) is suppressed. Activating the insulin receptor and GLUT‐4 together triggers the insulin signaling cascade and reverses insulin resistance (Jayaraman et al., 2021). In the type‐2 diabetic rat's model, insulin receptor molecule activation enhanced the translocation of GLUT4 inside the skeletal muscle, and β‐sitosterol reverses hyperglycemia and lowers the symptoms associated with type‐2 diabetes (Krishnan et al., 2021). They also suggested that to understand the antidiabetic effect of β‐sitosterol fully, it is also necessary to examine the capability of β‐sitosterol on the expression of additional insulin signaling molecules, including insulin rceptor substrate 1 (IRS‐1), insulin receptor substrate 2 (IRS‐2), and Akt. Afifi et al. (2022) claim that the β‐sitosterol glucoside‐loaded nanosystems (self‐nanoemulsifying drug delivery systems (SEDDS)) have the potential to slow the progression of diabetes in streptozotocin‐induced hyperglycemic rats by reducing insulin resistance, protecting against oxidative stress, and restoring pancreatic β‐cell secretory function. Notably, β‐sitosterol‐loaded nanosystems decreased serum glucose (63.22%), insulin (53.11%), and malondialdehyde (MDA) (38.31%) and increased catalase levels (64.45%). A high‐fructose diet was given to the rats, and after confirming diabetes, the oral route supplement of β‐sitosterol (25 mg/kg body weight) was given every day during a 30‐day trial until the finish of the study (Sekar et al., 2022). Rats with diabetes had lower amounts of glycogen, I.R., and GLUT4 insulin signaling proteins and less glucose and insulin tolerance. It was shown that diabetic rats had increased serum insulin levels. The use of β‐sitosterol in the treatment kept the blood sugar, serum insulin, and I.R. and GLUT4 protein levels normal. It proved that type‐2 diabetic rats given a high‐fat diet and activated I.R. and GLUT4 in the quadriceps muscle by β‐sitosterol improves glycemic control. β‐Sitosterol antidiabetic effects are summarized in Table 4 and discussed in detail below.

**TABLE 4:** Summary of research on the effects of β‐sitosterol in diabetes treatment, both in vitro and in vivo.

S. No.	Animal model	Dose, duration, and route of administration	Main findings	References
1	Streptozotocin‐induced diabetic rats	β‐Sitosterol (10, 15, and 20 mg/kg b.wt/day), orally for 21 days	Administration of β‐sitosterol reduces glycated hemoglobin, serum glucose, and nitric oxide and is also associated with upsurge in serum insulin. Pancreatic antioxidant levels were elevated by β‐sitosterol, accompanied by a reduction in compounds reactive to thiobarbituric acid	Gupta et al. (2011)
2	Alloxan‐induced diabetic rats	β‐Sitosterol (100 and 200 mg per kg b.wt/day), orally for 28 days	β‐Sitosterol stimulated the production of insulin via restoring the β‐cells. The assessment of insulin levels, the anti‐hyperglycemic effects, and the investigation of different metabolic enzymes involved in the pathways all showed that the increased insulin levels positively upheld the activities of glucose use and the glycogen storage pathways	Ramu et al. (2016)
3	High‐fat diet/sucrose‐induced type‐2 diabetic rats	Oral administration of β‐sitosterol (5, 10, 20, and 30 mg/ kg b.wt/day) for 30 days	By regulating insulin signaling molecules (I.R. and GLUT4), β‐sitosterol has shown anti‐hyperglycemic and anti‐hyperlipidemic effects in diabetic rats. It also normalizes biochemical parameters, oxidative stress markers, and the activity of antioxidant enzymes	Ponnulakshmi et al. (2019)
4	High‐fat diet/Fructose fed type‐2 diabetic rats	Oral supplement of β‐sitosterol (20 mg/ kg b.wt/day) for 30 days	The level of H_2_O_2_, OH, and LPO in the liver of diabetic rats was increased compared to the control, whereas administration of β‐sitosterol controls the increase of H_2_O_2_, OH, and LPO in the liver	Akilarooran et al. (2019)
5	High fat diet fed type‐2 diabetic rats	Oral administration of β‐sitosterol (20 mg/ kg b.wt/day) for 30 days	Diabetic rats showed reduced adiponectin levels and an upsurge in leptin levels, while treatment with β‐sitosterol considerably restored the altered levels in these animals	Chandran et al. (2019)
6	High‐fructose diet‐induced metabolic dysfunction	Oral supplement of β‐sitosterol (20 mg/ kg b.wt/day), orally for 12 weeks.	β‐Sitosterol prevented visceral obesity, hypertriglyceridemia, and hypoadiponectinemia brought on by a high‐fructose diet. It raised plasma adiponectin levels while lowering plasma insulin levels and the homeostatic model assessment index	Gumede et al. (2020)
7	High‐fat diet /Streptozotocin‐induced diabetic rats	β‐Sitosterol (5, 10, and 15 mg/kg b.wt/day), orally for 21 days	Administration of β‐sitosterol helped control excessive water and food consumption and weight loss in diabetic rats. However, insulin, hemoglobin, and protein expression (PPARc and GLUT4) significantly increased in insulin‐dependent tissues. Furthermore, β‐sitosterol lowered the plasma glucose and glycosylated hemoglobin	Ramalingam et al. (2020)
8	High‐fat diet/sucrose‐induced type‐2 diabetic rats	β‐Sitosterol (20 mg/ kg b.wt/day), orally for 30 days	By reducing inflammation in adipose tissue and obstructing the IKKβ/NF‐κB and JNK signaling pathways, β‐sitosterol prevents obesity‐induced insulin resistance	Jayaraman et al. (2021)
9	High‐fat diet/sucrose‐induced type‐2 diabetic rats	β‐Sitosterol (5, 10, 20, and 30 mg/kg b.wt/day), orally for 30 days	In diabetic rats, elevated levels of serum insulin, lipid profile, LPO, H_2_O_2_, and O.H.* have been observed. Supplement of β‐sitosterol stabilized changed levels of GLUT4 protein, blood sugar, serum insulin, lipid profile, and oxidative stress markers	Krishnan et al. (2021)
10	Streptozotocin‐induced diabetic rats	β‐Sitosterol glucoside (B.S.)‐loaded self‐nanoemulsifying drug delivery systems (SEDDS)	In addition to lowering hyperglycemia, BS‐SEDDS reduces the inflammation in the pancreas compared to the control. As a result, it can stop the progression of insulin resistance	Afifi et al. (2022)
11	High‐fat diet‐induced type‐2 diabetic rats	β‐Sitosterol (25 mg/ kg b.wt/day), orally for 30 days	Diabetic rats showed decreased levels of insulin signaling proteins GLUT4 and I.R., glycogen, and glucose and insulin tolerance. Serum insulin levels in diabetic rats were reported to be higher. When β‐sitosterol was used in the treatment, the levels of I.R. and GLUT4 protein, blood sugar, and serum insulin remained normal	Sekar et al. (2022)

## 7 CONCLUSIONS AND FUTURE PERSPECTIVE

β‐Sitosterol is a naturally occurring substance abundant in food and is neither mutagenic nor genotoxic. Several in vitro and in vivo investigations on the preventive effects of β‐sitosterol against cancer and diabetes have produced encouraging results. These preventive effects resulted via regulating the multiple pathways and various adaptive mechanisms. Though, the adage “there is always room for improvement” well captures the progress rate to advance β‐sitosterol as a promising therapeutic agent. Hence, numerous challenges, unanswered questions, and obstacles must be solved before β‐sitosterol can be extensively used as an effective treatment for human diseases. We start by discussing the obstacles to β‐sitosterol absorption, bioavailability, and solubility that restrict the therapeutic application and clinical use. Researchers showed that it has a low intestinal absorption capacity and a high rate of biliary excretion; therefore, β‐sitosterol bioavailability is low. And so, rather than manifesting systemically, the biological action of β‐sitosterol is expected to be localized in the lumen of the large bowel. Although, the administration route and carriers are challenging due to the β‐sitosterol's limited solubility in water. Several β‐sitosterol nanoformulations have been developed recently to enhance the β‐sitosterol water solubility, bioavailability, and targeting capacity. These β‐sitosterol nanoformulations have been tested for their ability to combat various human diseases and have more encouraging results. In light of the research data discussed in this review, some of the β‐sitosterol nanoformulations obstruct multiple pathways associated with the progress of cancer and diabetes. However, further study should be done to increase the β‐sitosterol efficacy using contemporary drug delivery systems, and more studies should be planned better to understand the anticancer and antidiabetic mechanisms of β‐sitosterol. So far, studies conducted using β‐sitosterol or β‐sitosterol nanoformulations have been performed on preclinical models. Therefore, a major drawback is our limited understanding of the risks and side effects of β‐sitosterol administration in humans. The compelling results from in vitro and in vivo research pave the way to design clinical studies and help to thoroughly comprehend the β‐sitosterol protective effect against human diseases, including cancer and diabetes. Therefore, it is important and urgent to conduct randomized controlled clinical trials with an adequate number of focused, driven, and well‐organized patients to assess the efficacy of β‐sitosterol as a protective therapeutic agent to treat cancer and diabetes. So far, using different carrier materials, researchers have developed several β‐sitosterol nanoformulations, but they are not tissue‐specific. Therefore, it is possible that β‐sitosterol nanoformulations delivered into healthy tissues present near cancer cells. Therefore, additional focus may be placed on generating tissue‐specific nanodrug delivery systems. It is also important to look into whether β‐sitosterol can be employed as a drug on its individual or in a suitable combination with another drug, which could increase its potential for the cutting edge of therapeutic approaches. And finally, the use of β‐sitosterol is still in its early stages. Developing β‐sitosterol as a therapeutic agent for treating diabetes and cancer requires continued research and dedicated efforts through well‐planned and scheduled trials.

## AUTHOR CONTRIBUTIONS

**Karthikeyan Adhimoolam:** Conceptualization (lead); funding acquisition (lead); investigation (lead); project administration (equal); writing – original draft (lead); writing – review and editing (lead). **Anjana Sureshbabu:** Writing – review and editing (supporting). **Elena Smirnova:** Writing – review and editing (supporting). **Pandiyan Muthuramalingam:** Writing – original draft (supporting); writing – review and editing (supporting). **Cat Tuong Do Thi:** Writing – review and editing (supporting). **Kalaiselvi Senthil:** Supervision (lead). **Taesun Min:** Conceptualization (supporting); project administration (equal); supervision (lead).

## FUNDING INFORMATION

This work was supported by the Creative Challenge Program (2020R1|1A1A01060923) to Adhimoolam Karthikeyan (A.K.) and the Basic Science Research Program (2019R1A6A1A11052070 and 2022R1A2B5B02001711) to Taesun Min (T.M.) through the National Research Foundation of Korea (NRF), Ministry of Science and ICT.

## CONFLICT OF INTEREST STATEMENT

The authors declare that they have no known competing financial interests or personal relationships that could have appeared to influence the work reported in this paper.

## CONSENT FOR PUBLICATION

All authors have read and agreed to the published version of the manuscript. All authors read and approved the final manuscript.

## Data Availability

No data were used for the research described in the article.
